# Addressing widespread detection heterogeneity in avian occupancy modeling using passive acoustic surveys

**DOI:** 10.1093/ornithapp/duag006

**Published:** 2026-01-22

**Authors:** Tessa A Rhinehart, Chapin Czarnecki, R Patrick Lyon, Lauren M Chronister, Sam Lapp, Jeffery L Larkin, Jeffery T Larkin, Darin J Mcneil, Jacob Goldman, Justin Kitzes

**Affiliations:** Department of Biological Sciences, University of Pittsburgh, Pittsburgh, Pennsylvania, USA; Department of Biological Sciences, University of Pittsburgh, Pittsburgh, Pennsylvania, USA; Department of Biological Sciences, University of Pittsburgh, Pittsburgh, Pennsylvania, USA; Department of Biological Sciences, University of Pittsburgh, Pittsburgh, Pennsylvania, USA; Department of Biological Sciences, University of Pittsburgh, Pittsburgh, Pennsylvania, USA; Department of Biology, Indiana University of Pennsylvania, Indiana, Pennsylvania, USA; American Bird Conservancy, The Plains, Virginia, USA; Department of Environmental Conservation, University of Massachusetts Amherst, Amherst, Massachusetts, USA; Department of Forestry and Natural Resources, University of Kentucky, Lexington, Kentucky, USA; Department of Biology, Indiana University of Pennsylvania, Indiana, Pennsylvania, USA; Department of Biological Sciences, University of Pittsburgh, Pittsburgh, Pennsylvania, USA

**Keywords:** autonomous recording units, detection heterogeneity, occupancy modeling, passive acoustic monitoring, point counts, within-territory movement, conteos por puntos, heterogeneidad de detección, modelado de ocupación, monitoreo acústico pasivo (MAP), movimiento dentro del territorio, unidades de grabación autónomas

## Abstract

Ornithologists have embraced occupancy models to account for imperfect species detection. These models misestimate occupancy when they fail to model detection heterogeneity, variability in the probability of species detection. Practitioners typically account for detection heterogeneity using site and survey covariates, but generally ignore heterogeneity arising from ubiquitous processes like within-territory movement and territory position relative to the observer. Furthermore, while such heterogeneity may be widespread, traditional in-person monitoring projects usually lack the temporal resolution to quantify it. Passive acoustic monitoring (PAM) provides an alternative, enabling collection of more visits per site for less field effort. We investigated how spatial processes create detection heterogeneity, how the number of visits per site impacts diagnosis of heterogeneity, and how heterogeneity biases occupancy estimates for 3 occupancy modeling approaches. We simulated territory position, within-territory movement, and auditory detections of individual birds to generate single-season detection histories with up to 20 visits per site for varying avian occupancy probabilities, densities, and territory sizes. We also collected 3 large-scale single-season PAM datasets containing 10–21 visits per site. We modeled occupancy using the “basic” single-season, zero-inflated binomial occupancy model, the Royle-Nichols (RN) abundance model, and the zero-inflated beta-binomial (ZIBB) model, which draws site detection probability from a beta distribution. Detection heterogeneity was common in simulations across realistic ranges of density and territory size. Diagnosing heterogeneity required 3–20 visits per site, depending on occupancy probability and severity of heterogeneity. Heterogeneity caused occupancy misestimations from 94% underestimates to 400% overestimates. The RN model’s estimates were the most biased. The basic and ZIBB models produced negligibly biased estimates with increasing numbers of visits, the ZIBB model requiring fewer visits. Our results suggest widespread spatial processes may bias occupancy estimation from point-count and PAM surveys, but many-visit detection histories generated by PAM improve diagnosis of detection heterogeneity and accuracy of occupancy estimates.

## RESUMEN

Los ornitólogos han adoptado los modelos de ocupación para tener en cuenta la detección imperfecta de las especies. Estos modelos estiman incorrectamente la ocupación cuando no modelan la heterogeneidad de detección, es decir, la variabilidad en la probabilidad de detectar una especie. Quienes aplican estos modelos suelen considerar la heterogeneidad de detección mediante covariables del sitio y del muestreo, pero generalmente ignoran la heterogeneidad que surge de procesos omnipresentes como el movimiento dentro del territorio y la posición del territorio en relación con el observador. Además, aunque dicha heterogeneidad puede ser generalizada, los proyectos tradicionales de monitoreo presencial suelen carecer de la resolución temporal necesaria para cuantificarla. El monitoreo acústico pasivo (MAP) ofrece una alternativa al permitir obtener más visitas por sitio con menos esfuerzo de campo. Investigamos cómo los procesos espaciales generan heterogeneidad de detección, cómo el número de visitas por sitio influye en el diagnóstico de dicha heterogeneidad y cómo la heterogeneidad sesga las estimaciones de ocupación considerando tres enfoques de modelado de ocupación. Simulamos la posición del territorio, el movimiento dentro del territorio y las detecciones auditivas de aves individuales para generar historiales de detección para una sola temporada con hasta 20 visitas por sitio, para distintas probabilidades de ocupación, densidades y tamaños de territorio. También recopilamos tres conjuntos de datos a gran escala de MAP para una sola temporada con 10–21 visitas por sitio. Modelamos la ocupación usando el modelo “básico” de ocupación binomial con exceso de ceros para una sola temporada, el modelo de abundancia de Royle-Nichols (RN) y el modelo beta-binomial con exceso de ceros (BBEC), que extrae la probabilidad de detección del sitio de una distribución beta. La heterogeneidad de detección fue frecuente en las simulaciones en rangos realistas de densidad y tamaño territorial. Diagnosticar la heterogeneidad requirió entre 3 y 20 visitas por sitio, según la probabilidad de ocupación y la severidad de la heterogeneidad. La heterogeneidad provocó errores en las estimaciones de ocupación que variaron desde subestimaciones del 94% hasta sobreestimaciones del 400%. Las estimaciones del modelo RN fueron las más sesgadas. Los modelos básico y BBEC produjeron estimaciones con sesgos insignificantes a medida que aumentaba el número de visitas, siendo el modelo BBEC el que requirió menos visitas. Nuestros resultados sugieren que los procesos espaciales generalizados pueden sesgar las estimaciones de ocupación basadas en conteos por puntos y en MAP, pero que los historiales de detección con muchas visitas generados por MAP mejoran el diagnóstico de la heterogeneidad de detección y la precisión de las estimaciones de ocupación.

## INTRODUCTION

Animal monitoring programs frequently fail to detect target species at some sites where the species is present (Kellner and Swihart 2014). When nondetection occurs, monitoring programs may underestimate population sizes or ranges and misestimate relationships between animals and their environments (Gu and Swihart 2004, Kéry et al. 2010, Guillera-Arroita 2017, Lahoz-Monfort and Magrath 2021). If unaccounted for, nondetection can undermine the comparability of monitoring programs and may ultimately result in the failure to identify and protect critical habitat (Sólymos et al. 2013, Bennett et al. 2024). Many practitioners monitoring patterns of occupancy and abundance account for nondetection using hierarchical models that model monitoring data as arising from a process of imperfect observation of the true occupancy or abundance. Occupancy models, which estimate the proportion of sites occupied by the target species (MacKenzie et al. 2002, 2017), are among the most frequently used in part because they rely on detection-nondetection data, which are widely available and relatively straightforward to collect.

Occupancy models make the key assumption that there exists no unmodeled variability in detection probability across sites and visits. In particular, standard single-season occupancy models assume detection probability is either constant across sites and surveys or is constant conditional on the effects of a set of modeled detection covariates (MacKenzie et al. 2017). Variability in the survey-level probability of detecting a species at an occupied site, also known as detection heterogeneity, is a principal area of concern for practitioners using occupancy modeling (Kellner and Swihart 2014). Most applications of occupancy modeling attempt to account for detection heterogeneity using site- and visit-level covariates hypothesized to impact detection, such as site attributes (e.g., shrub density or road exposure; Anderson et al. 2015, Cooke et al. 2019), survey conditions (e.g., date, time of day, weather, or background noise; Bas et al. 2008, Pacifici et al. 2008), and differences in observer skill (e.g., Miller et al. 2015, Schmidt et al. 2023). Unmodeled detection heterogeneity can undermine the ability to assess population status and relationships with environmental covariates accurately. Unmodeled heterogeneity is known to result in underestimation of occupancy probability (Royle and Nichols 2003, Royle 2006, Schmidt et al. 2023) or in overconfident or spurious estimates of site and survey covariates (McClintock et al. 2010a, DiRenzo et al. 2022). When site-level variation in detection probability is not fully explained by covariates or otherwise modeled, occupancy is typically underestimated (the “second law of capture–recapture;” see Royle 2006, Kéry and Royle 2016, pp. 560–561). Underestimates arise from the species being detected more frequently at sites with higher detection probabilities. This overrepresentation of detections from sites where detection probability is higher leads to an overestimation of the average detection probability and commensurate underestimation of the occupancy probability (Laake and Collier 2024).

In addition to arising from differences in detectability across site or survey conditions, detection heterogeneity can arise from widespread, random spatial processes, such as movement of individual animals within their home ranges and random positioning of the survey point with respect to individuals’ positions. The position of the survey point creates an arbitrary sampling area in otherwise continuous habitat that does not necessarily correspond to the boundaries of individuals’ home ranges (Efford and Dawson 2012). Movement of individuals within their home ranges may cause temporary emigration out of the effectively surveyed area, by which an individual becomes periodically unavailable for detection (e.g., by moving to a part of its range where it is effectively undetectable; Chandler et al. 2011, Efford and Dawson 2012). Temporary emigration can induce uneven site detection probabilities due to varying amounts of overlap between the home range of an individual and the area in which a surveyor can detect it (Nichols et al. 2009, DiRenzo et al. 2022). Even without complete emigration, movement within the home range that changes the distance of an individual from a survey point impacts the probability of detecting the individual from visit to visit (Simons et al. 2007, Efford and Dawson 2009, McClintock et al. 2010b, Buckland et al. 2015). Furthermore, because the position of each individual’s home range relative to the observer varies randomly from site to site, detection probability may also differ between sites even if a given individual never exits the survey area.

A variety of modeling approaches exist to account for detection heterogeneity unrelated to covariates, though none are perfect. Multi-scale occupancy models with temporal replication across sites can account for temporary emigration by explicitly modeling availability for detection as a stochastic process (Mordecai et al. 2011, DiRenzo et al. 2022), though they require a third layer of survey replication (e.g., subdivision of surveys into minute-by-minute counts) that may limit their application. The Royle and Nichols (2003) model of abundance makes the assumption that each individual has an equal and independent probability of detection, thereby modeling site-level detection heterogeneity that arises due to differences in animal abundance, although its flexibility may be limited by its assumption of constant per-individual detectability given covariates. Notably, when point-count protocols include distance estimation, distance sampling techniques can account for heterogeneity caused by the distance between individuals and the observer (Buckland et al. 2015), and can account for temporary emigration using temporal replication (Chandler et al. 2011) and individual movement by using animal telemetry data (Glennie et al. 2021). Yet distance estimation errors are common and likely generate considerable heterogeneity (e.g., Alldredge et al. 2007, Nadeau and Conway 2012), even when the amplitude of vocalizations can be quantified from calibrated acoustic recordings. To relax the assumption of uniform detection probability across sites, when the heterogeneity cannot be explained by covariates, site-level detection probability can be modeled as a draw from a statistical distribution (e.g., the beta distribution, Royle 2006).

**FIGURE 1.**
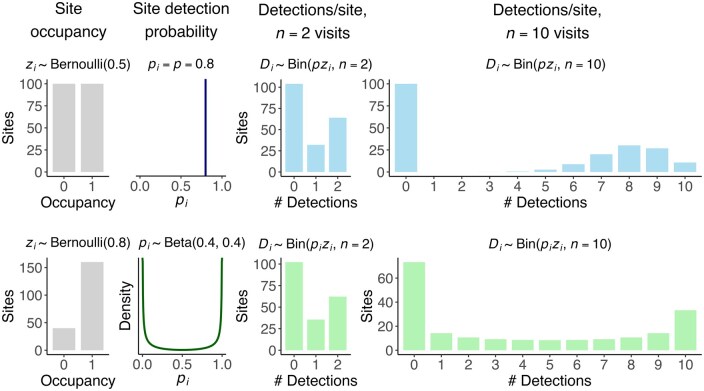
Short detection histories can mask detection heterogeneity and make occupancy estimation more challenging. The first row shows a simulated distribution of detections per site on a landscape with 50% of sites occupied and a constant detection probability p=0.8. The second row shows a simulated distribution of detections per site in a landscape with 80% of sites occupied, where the detection probability at a site i is drawn from a beta distribution, $p_{i}\;∼$ Beta (α=β=0.4). With only two visits to each site, the expected distributions of detections per site of the two rows are highly similar, and a basic occupancy model estimates that the two landscapes had nearly identical percentages of occupied sites (50% and 52%, respectively). At 10 visits per site, the differences between landscapes in the distributions of detections per site are apparent, although a basic occupancy model still underestimates the percentage of occupied sites in the second landscape (64%).

Researchers designing surveys for occupancy modeling often face a trade-off between the number of sites to survey and the number of visits per site (MacKenzie and Royle 2005, Bailey et al. 2007). While the tendency may be to opt for greater spatial replication, fewer visits may limit not only the ability to estimate detection probability (Guillera-Arroita et al. 2010), but also the ability to identify and estimate detection heterogeneity when it occurs. To illustrate this possibility, consider the two landscapes illustrated by the two rows in Figure 1. In the first landscape, 50% of sites are occupied, and detection probability is 0.8 (vertical line). In the second landscape, 80% of sites are occupied, a markedly higher percentage than in the first landscape. In the second landscape, detection probability varies by site according to a beta distribution; most sites are expected to have either a high detection probability near 1 (for example, survey plots overlapping the core of an individual’s home range) or a low detection probability near 0 (e.g., survey plots where the bird’s home range is on the margin of the survey area), with few sites expected to have intermediate detection probability. Despite having different occupancy probabilities and detection processes, when only 2 visits are conducted per site, these landscapes have nearly identical expected distributions of the number of detections per site (i.e., the number of sites with a detection on 0, 1, or 2 visits). Thus, models not designed to account for detection heterogeneity without covariates would have little power to distinguish these two scenarios. In the first landscape with constant detection probability, a basic occupancy model accurately estimates occupancy as 50% of sites occupied, but in the second landscape with unmodeled detection heterogeneity, the model underestimates occupancy at 52% when true occupancy is 80%. With 10 visits per site, in contrast, the difference in the landscapes’ distributions of detections per site becomes apparent, although in the second landscape, a basic occupancy model still underestimates occupancy at 64%.

While there is reason to believe individual space use and within-territory movement generate detection heterogeneity, few studies investigate how commonly this happens, how readily it can be diagnosed, and how it impacts occupancy estimates. First, it is challenging to identify how widespread this source of heterogeneity is using typical detection-nondetection data. While detection heterogeneity can be apparent in detection histories with many visits (e.g., Figure 1), other phenomena could generate similar patterns, such as closure violations from territory shifts or abandonment (e.g., Rota et al. 2009, Valente et al. 2024). Distinguishing patterns generated by territory shifts or abandonment from those generated by within-territory movement would require more detailed data than is typically available (e.g., spatially replicated individual home range mapping). Second, goodness-of-fit tests may aid in the diagnosis of detection heterogeneity or poor model fit, but it is unknown whether these tests have the power to diagnose realistic levels of detection heterogeneity unrelated to covariates, in particular in light of the number of visits per site, occupancy probability, and availability. Third, while unmodeled detection heterogeneity is well-known to cause biased occupancy estimates, it is unknown whether realistic levels of individual space use and movement generate enough heterogeneity to bias occupancy estimation.

We use simulated and field data to understand (1) how random spatial processes generate detection heterogeneity, (2) how the number of repeat visits per site impacts the ability to diagnose poor model fit to detection histories, and (3) how heterogeneity and the number of visits per site impact occupancy estimation. We built on prior agent-based simulations of binary temporary emigration (occupancy modeling, Hayes and Monfils 2015; count modeling, Chandler et al. 2011) by modeling the detectability process as it occurs on a point count, allowing the distance of an individual animal from a survey point to impact its detectability continuously. We used *bSims* (Sólymos 2023), an agent-based point count simulator, to simulate single-season, 20-visit detection histories in the presence of individual movement. We parameterize our simulations across a broad range of realistic territory sizes, population densities, and landscape-level occupancy probabilities. We also collected detection histories with at least 10 visits per site from the field using passive acoustic monitoring (PAM), which can survey many days in a row while only conducting 2 in-person visits to each site (recorder dropoff and pickup). We include field data with detection histories ranging from 10 to 21 visits per site for 3 declining bird species: a songbird (*Hylocichla mustelina*, Wood Thrush), a nightjar (*Antrostomus vociferus*, Eastern Whip-Poor-Will), and a shorebird (*Scolopax minor*, American Woodcock).

For each of our datasets, we modeled occupancy using a basic binomial occupancy model, the Royle beta-binomial model (Royle 2006), and the Royle-Nichols abundance model (Royle and Nichols 2003). We determined how many datasets had site detection heterogeneity (non-constant detection probability across sites) by testing for deviation of the distribution of the number of detections per site from the binomial distribution. We then evaluated each model’s fit to each detection history by testing whether the predicted distribution of detections significantly deviated from the observed distribution. We explored how the number of visits per site impacted our ability to diagnose detection heterogeneity and poor model fit. Finally, we investigated the extent to which detection heterogeneity biased occupancy estimates by each of the 3 models.

## METHODS

### Simulated Data

We simulated 20-visit detection histories for scenarios we hypothesized could produce detection heterogeneity not explainable by site or survey-level covariates. In particular, we investigated the impacts of within-territory movement of individual birds and random positioning of territories on the landscape at a given constant density, not associated with habitat covariates. We accomplished this using *bSims* 0.3-2 (Sólymos 2023), an agent-based point count simulator in R 4.4.0 (R Core Team 2024), which produces simulated detection histories using realistic assumptions about bird movement and the detection process. We designed our detection histories to be comparable to the detection process of PAM with manually vetted detections, or to that of auditory-only point counts.

We simulated detection histories for 200 sites for each of 90 scenarios representing combinations of parameters for density (6 levels), territory size (5 levels), and site occupancy (3 levels). We generated 100 simulations for each of the 90 scenarios, resulting in a total of 9,000 simulations. We used *bSims* to simulate 20-visit detection histories for occupied sites. First, for each occupied site, we populated a landscape with individual birds’ territory centers at the chosen density. We then animated individuals’ within-territory movement and production of auditory cues at the chosen territory size. Finally, to simulate the PAM detection process, we simulated the detection of auditory cues only. We simulated 20 independent visits to each population of individuals by repeating the animation and detection processes 20 times.

Each occupied site was simulated by populating a separate 1.5 × 1.5 km landscape. Within a given scenario, density did not vary between occupied sites due to environmental covariates, so detection heterogeneity from density arose due to the stochastic placement of individual birds’ territories, rather than differences in the underlying population density between sites. Territory centers (“nest” in *bSims*) were stochastically placed with semi-regular spacing (*sensu*  Sólymos 2023), such that territory centers were unlikely to be close to each other, as is typical of territorial songbirds, which tend to defend non-overlapping territories (Askins 1987, e.g., Zach and Falls 1979, Frantz et al. 2016). Within a given scenario, we populated all occupied sites with 1 of 5 simulated density levels of 0.1 individuals, 0.25 individuals, 0.5 individuals, 1 individual, and 2 individuals per hectare. The number of detectable individuals at each site depended on the random positioning of individual territories with respect to the observer. To complement these simulations, we included a sixth level of density in which we populated each occupied site with exactly one detectable individual.

For each of the 20 visits to a given occupied landscape, we simulated within-territory movement as a stochastic process. The timing of individual bird movements followed an exponential distribution with a mean of one movement per minute. The position each individual moved to was drawn from a bivariate normal distribution centered on the individual’s territory center, where the SD of the two normal variables were identical, creating roughly circular “territories.” We varied the SD of the distribution (0 m, 10 m, 25 m, 50 m, and 100 m) to simulate a range of territory sizes and movement patterns, including no movement to simulate species that consistently display from more or less a single position (e.g., *S. minor*). We simulated movement of each individual only within its defended territory, not a larger home range (*sensu*  Odum and Kuenzler 1955); thus, each individual bird could produce an auditory cue from any of the simulated positions it moved to.

We simulated a “surveyor” at the center of each landscape conducting 20 separate 10-min detection-nondetection surveys per site, as point counts are commonly 10 min long (Ralph et al. 1995). Because our goal was to simulate the detection process of PAM with manually vetted detections, we assumed the surveyor only detected auditory cues; this assumption is reasonable for point counts as well, as over 95% of avian detections in forests are estimated to be auditory (Brewster and Simons 2009). We selected the 2 parameters governing birds’ detectability (i.e., their availability and perceptibility; Sólymos et al. 2013) to be approximately equal to the median estimate by Edwards et al. (2023) for point counts of 60 North American forest songbirds. Birds’ availability for detection was simulated using an exponential distribution with an average vocalization rate of 0.25 auditory cues min^−1^. Perceptibility of cues was simulated using a half-normal detection function with a 60-m effective detection radius, leading to the perceptibility of a cue to be about 97% at 10 m, 50% at 50 m, and 6% at 100 m. We also investigated how increasing perceptibility to an effective detection radius of 90 m and 120 m impacted detection heterogeneity (results presented in Supplementary Material Figure 1). Although perceptibility varies between point count and PAM protocols, and can be impacted by PAM data analysis techniques (e.g., performance of machine learning algorithms for species identification), we found that simulated detection histories were consistent with those observed in the field data (below) and were plausible from our personal experience with PAM. All other parameters were set to their default in *bSims*.

Occupancy is notably challenging to define for moving animals when sampling a subset of a continuous habitat, as is typical of point counts and PAM surveys (Efford and Dawson 2012, Wood and Peery 2022). In particular, individual animals may only use a small fraction of the sampled area, and much of an individual’s home range may be outside of the sampled area. We considered a site occupied if the core of at least one individual’s territory at least partially overlapped the sampling range of the observer (Nichols et al. 2009). We defined an individual’s core territory as the circle with radius equal to 2 SD of movement around the territory center, in which 86% of the bird’s movements were expected to fall. Thus, the sizes of core territories for each SD of movement were 0 ha, 0.13 ha, 0.7 ha, 3.1 ha, and 12.57 ha, respectively. We defined the sampling range as a circle around the observer for which a single vocalization produced at the outer edge of the circle had a higher than 6% probability of detection, corresponding to a 100 m radius for our simulations. To ensure each occupied site could reasonably be considered to have at least one detectable individual using the site, we repeated simulations until the core of at least one individual’s territory at least partially overlapped the sampling range. Altogether, this approach ensured that every occupied site contained at least one individual expected to have ∼1% or more of its movements fall within 100 m of the surveyor.

For each simulation, we simulated 200 sites, for which either 20%, 50%, or 80% of the sites were occupied (40, 100, or 160 sites). To reduce the computational resources required to generate our data, we simulated 160 occupied sites for each combination of territory size and density. We then selected either 40 or 100 of the simulated occupied sites to achieve lower levels of realized occupancy. The remaining sites were unoccupied and were represented in the detection history as sites without detections during any visit. Simulation of 9,000 detection histories completed in ∼44 hours on 10 processing cores.

### Field Data

As real-world examples of many-visit detection histories, we used field data from passive acoustic monitoring projects to monitor 3 species: (1) *H. mustelina* in central and western Pennsylvania for 10 visits per site across 289 sites; (2) *A. vociferus* across the eastern U.S. for 16 visits per site across 350 sites; and (3) *S. minor* across Pennsylvania for 21 visits per site across 192 sites. For further description of the projects from which these field data originated, see the Methods in the Supplementary Materials.

All field data were collected by autonomous recorders and scored by machine learning classifiers to predict the presence of the species of interest in each 3–5 s clip of audio. For one data source (*A. vociferus*), the classifier achieved 100% precision (i.e., no false-positive predictions) and 35% recall on a test set at a given score threshold (Larkin et al. 2024). Thus, for this data source, we generated a detection history where a site had a “detection” on a given day if any clip recorded at the site on that day scored above this threshold. Otherwise, the site and day received a “nondetection.”

For the other data sources, clips receiving high scores from the machine-learning classifiers were manually reviewed by experienced listeners to verify the presence of the species. This was necessary because the classifiers for the remaining species produced non-negligible amounts of false-positive predictions. This procedure, “classifier-guided listening,” uses classifiers to guide verification efforts by showing listeners the clips that the classifier was most confident contained the species of interest on each site and day (Chronister et al. 2025). For two data sources, a given number of the high-scoring clips for each site and day were reviewed by a listener (number of clips reviewed per site-day: *S. minor *= 5, *H. mustelina *= 1). More details about the classifiers used and the classifier-guided listening approach are provided in the Methods in the Supplementary Materials.

### Occupancy Models

We fit 3 types of occupancy models to detection histories from our simulated and field data: (1) a basic single-season occupancy model with constant detection probability, (2) an occupancy model with a beta distribution on detection, and (3) the Royle-Nichols abundance estimation model, where detection probability is modeled to vary with the number of individuals per site (Royle and Nichols 2003).

#### Zero-inflated binomial (basic) model

The most widely used occupancy model is a single-season, zero-inflated binomial model, which we term the “basic” model. This model assumes for M sites, given some probability of occupancy ψ, that $z_{i}$, the occupancy state of site i, is Bernoulli distributed,


$$z_{i}\;∼\;\mathrm{Bernoulli}(\psi ),$$

where ψ is often modeled with environmental covariates (i.e., site covariates). The basic model is applied to detection-nondetection data collected during N visits per site, recording a detection ($y_{ij}=1$) or nondetection ($y_{ij}=0$) for each site i during each survey j.

At unoccupied sites ($z_{i}=0$), the species is assumed to never be detected (i.e., no false positive detections), whereas at occupied sites ($z_{i}=1$), the species is assumed to be detected at a site i on visit j according to a Bernoulli trial with probability p,


$$y_{i,\;j}\;∼\;\mathrm{Bernoulli}(z_{i}p),$$

where p is often modeled with site- and visit-level covariates, $p_{i,\;j}$. In the case where detection probability is constant across all visits at occupied sites, the number of detections $D_{i}$ of the species at an occupied site i over the course of the N visits is binomially distributed,


$$D_{i}\;|\;\mathrm{site}\;i\;\mathrm{occupied}\;∼\;\mathrm{Binomial}(n=N,\;p=p).$$

We assume unoccupied sites do not produce detections, such that the distribution of the number of detections per site across both occupied and unoccupied sites is a zero-inflated Binomial distribution with zero-inflation rate 1-ψ (Royle 2006),


$$D_{i}\;∼\;\mathrm{Zero}‐\mathrm{inflated}\;\mathrm{Binomial}(n=N,\;p=p,\;r=1-\psi ).$$

We fit the standard zero-inflated Binomial occupancy models using the *unmarked* package (Fiske and Chandler 2011, Kellner et al. 2023) in R.

#### Zero-inflated beta-binomial (ZIBB) model

We compared 2 alternative occupancy models that could be used for non-binomial detection histories (e.g., Figure 1). First, we used the beta-binomial occupancy model (Royle 2006). Unlike the basic model, which assumes that p is constant (or constant conditional on any parameters, when $p_{i,\;j}$ is modeled), this approach assumes detection probability varies on a per-site basis. Each site’s detection probability is drawn from a beta distribution, which has two parameters (α, β):


$$z_{i}\;∼\;\mathrm{Bernoulli}(\psi )$$

$$y_{i,\;j}\;∼\;\mathrm{Bernoulli}(z_{i}{\;p}_{i})$$

$$p_{i}\;∼\;\mathrm{Beta}(\alpha ,\;\beta ).$$

At an occupied site i, the number of detections $D_{i}$ of the species over the course of the N visits is distributed as a beta-binomial,


$$D_{i}\;|\;\mathrm{site}\;i\;\mathrm{occupied}\;∼\;\mathrm{Beta}‐\mathrm{Binomial}(n=N,\;p=p_{i},\;r=1-\psi ).$$

Incorporating unoccupied sites, the number of detections $D_{i}$ per site is distributed as a zero-inflated beta-binomial with zero-inflation rate 1-ψ,


$$D_{i}\;∼\;\mathrm{Zero}‐\mathrm{inflated}\;\mathrm{Beta}‐\mathrm{Binomial}(n=N,\;p=p_{i},\;r=1-\psi ).$$

We fit the zero-inflated beta-binomial (ZIBB) distribution to our data using maximum likelihood estimation with the ZIBB distribution provided by the VGAM package (v. 1.1-11, Yee 2024) in R.

#### Royle-Nichols (RN) model

The Royle-Nichols (RN) model was originally developed to model abundance by taking advantage of the detection heterogeneity arising from variation in abundance across sites (Royle and Nichols 2003). This approach models abundance at each site, $B_{i}$, as a latent variable estimated using detection-nondetection data:


$$B_{i}\;∼\text{Poisson}(\mathrm{\lambda ),}$$

where λ is an intensity parameter describing the density of animals across the landscape.

Each individual is assumed to have the same probability of detection on a survey, r. Thus, the probability of detection of at least one individual of the species at a given site i, termed $p_{i}$, is conditional on the number of individuals available for detection, $B_{i}$,


$$p_{i}=1-(1-r{)}^{B_{i}}.$$

Then, similar to the above models, the number of detections $D_{i}$ of the species at a site i over the course of the N visits is binomially distributed,


$$D_{i}∼\;\mathrm{Binomial}(n=N,\;p=p_{i}).$$

Occupancy estimates from this model are derived from the density estimate (λ) as one minus the probability that a site has no individuals, 1- Poisson(x=0 | λ).

We fit the RN model using the occuRN function in the *unmarked* package (Fiske and Chandler 2011, Kellner et al. 2023) in R.

#### Model fitting

We not only fit models to the full detection history $D_{i}$, but also to shortened detection histories T={2, 3, …, 20}. For the simulated data, we created shortened histories by selecting the first T visits from each detection history. For the field data, in order to reduce detection heterogeneity arising from visit date, we shuffled the order of visits for each site independently, then created shortened histories by selecting the first T visits from each shuffled detection history. Model fitting and goodness-of-fit tests for the 9,000 simulated datasets and three field datasets were completed in ∼7 hr total on 10 processing cores.

### Detection Heterogeneity Tests

If probability of detection is constant across occupied sites, the number of visits with a detection per occupied site, $D_{i}$ | site i occupied, is expected to be distributed binomially (see above). We diagnosed site-level detection heterogeneity in the underlying detection process by testing whether this distribution deviates from the binomial distribution. For simulated data, we fit a binomial distribution to full 20-visit detection histories from all occupied sites, including those with zero detections. For field data, where true occupancy status at sites without detections is unknown, we fit a zero-truncated binomial distribution to the detection history for the group of sites with at least one detection. For each dataset, we used the fit distribution to generate the distribution of detections per site that would be expected if the detection probability were constant.

We used Fisher’s exact test to assess whether the observed distribution of detections per occupied site deviated from the expected distribution, where lower *P*-values indicate greater strength of evidence of deviation. We considered the test to have identified detection heterogeneity in a dataset if P<0.05.

We applied the test to (1) identify which full-length, full-occupancy datasets contained a signature of detection heterogeneity, (2) assess whether particular simulated territory sizes and population density were associated with diagnosing detection heterogeneity in a greater proportion of simulations, and (3) assess how reducing the number of occupied sites (from 160 to 100 or 40) or number of visits (from 20 to 2–19) impacted the power to diagnose detection heterogeneity in simulated datasets where a signature of heterogeneity was identified in (1).

#### Goodness-of-fit tests

For each of the 9,000 simulated datasets and three field datasets, we also tested for poor model fit to detection histories under each of the 3 occupancy models using a test similar to that of MacKenzie and Bailey (2004). In particular, we used a Fisher’s exact test to assess whether the observed distribution of detections per site, $D_{i}$, significantly differed from the distribution expected by each fit occupancy model. This goodness-of-fit test differs from the detection heterogeneity test in two significant ways. First, the detection heterogeneity test only evaluates the basic occupancy model’s assumption of constant detection probability, whereas the goodness-of-fit test evaluates both the basic occupancy model structure and the more flexible structures of the other two occupancy models. Second, the detection heterogeneity test only assesses properties of data from occupied sites, whereas the goodness-of-fit test evaluates the structural assumptions and parameter estimates of models fit to data from both occupied and unoccupied sites. However, the goodness-of-fit test has low power to assess fit of the occupancy component (MacKenzie and Bailey 2004).

Because some values of $D_{i}$ are expected to have low frequencies, a traditional chi-squared goodness-of-fit test may not produce valid *P*-values, and an exact test is preferred (Kim 2017). Thus, we used Fisher’s exact test to produce valid *P*-values for the goodness-of-fit test. This approach was less computationally intensive than the commonly used parametric bootstrap approach described by MacKenzie and Bailey (2004). Lower *P*-values of the goodness-of-fit test indicate poorer model fit (i.e., greater deviation of the observed distribution $D_{i}$ from the expected distribution generated by a given fit model). We considered a model to fail the goodness-of-fit test if P<0.05.

We applied the test to (1) identify poor model fits, and (2) assess which values of simulated territory size, simulated population density, occupancy, and number of visits per site were associated with higher rates of failure of the goodness-of-fit test. We also assessed whether the *P*-value of the detection heterogeneity and goodness-of-fit tests were related to the magnitude of occupancy estimation error.

### Occupancy Estimation Accuracy

For the simulated datasets, we compared models’ estimated occupancy to the percentage of occupied sites included in the dataset, using the definition of occupancy above. Because our ad-hoc definition of occupancy makes models unlikely to recover the exact proportion of occupied sites, we assessed overall bias and variance in occupancy estimates arising from model misspecification. For the field datasets, true occupancy is unknown, as it is unknown how many sites were occupied but never received a detection. Thus, in our field datasets, we compared occupancy estimates to 2 quantities. First, we used sample-at-hand occupancy, the maximum naive occupancy across all visits in the dataset. The sample-at-hand occupancy provided a minimum known occupancy for the full dataset, enabling us to easily identify occupancy underestimates generated by models fit to subsamples of the dataset. Second, when we subset detection histories to a lower number of visits, we calculated naive occupancy, defined as the number of sites with at least one detection within that sample of visits. If a model’s occupancy estimate matches the naive occupancy, the model is estimating that there are no additional undetected occupied sites. When naive occupancy is lower than sample-at-hand occupancy, a model’s failure to estimate additional undetected occupied sites reflects an overestimation of detection probability. This is a commonly expected consequence of detection heterogeneity. We identified model failures (i.e., boundary estimates of occupancy near 0 or 1) and quantified how the proportion of failures varied by model and number of visits. In practice, we identified estimates > 0.99 as boundary estimates (Welsh et al. 2013).

## RESULTS

### Detection Heterogeneity Tests

**FIGURE 2.**
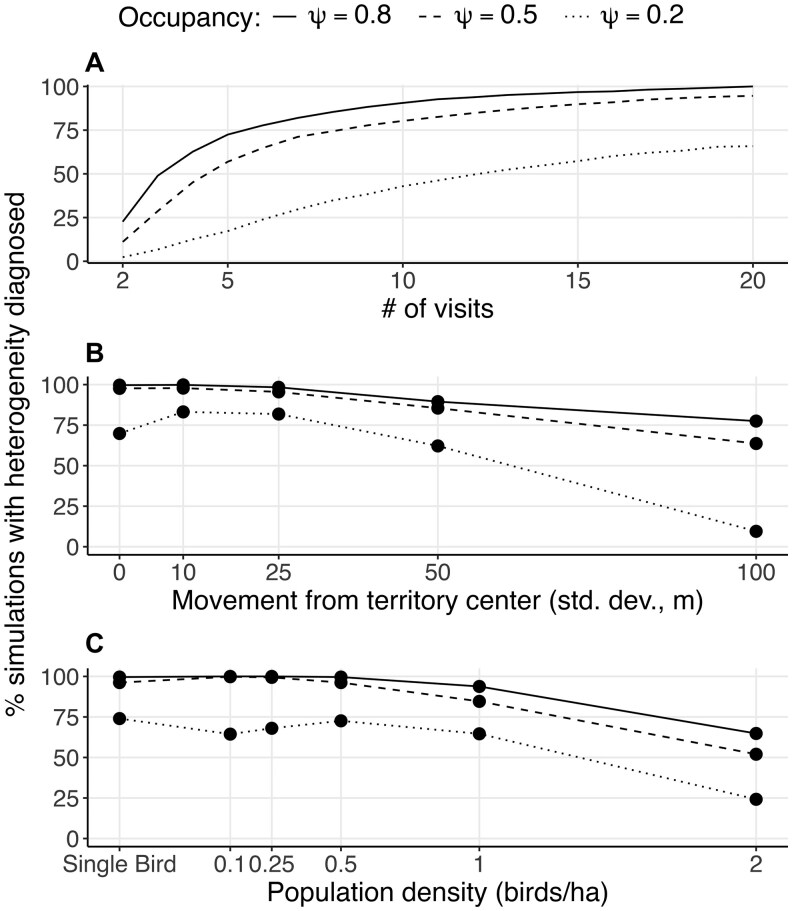
Detection heterogeneity is widespread, but it is more challenging to diagnose with fewer visits per site or lower occupancy probability. Each point represents the percentage of simulation scenarios diagnosed as having heterogeneous detection probabilities at each value of (**A**) number of visits per site, (**B**) SD of movement away from territory center (20-visit detection histories only), and (**C**) density of simulated population (20-visit detection histories only).

**FIGURE 3.**
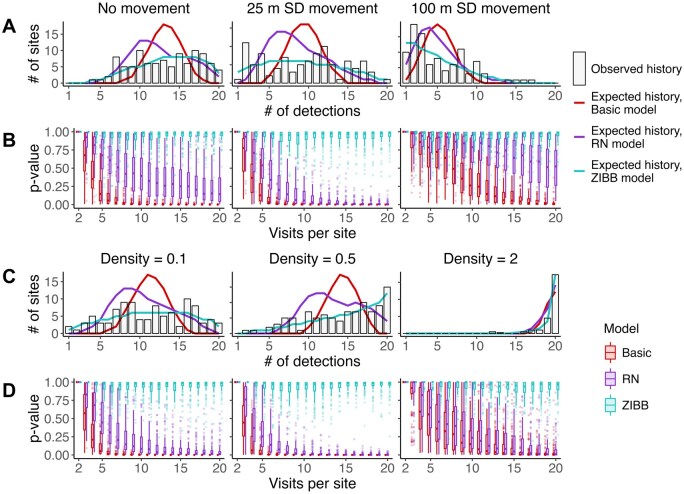
Detection heterogeneity leads to overdispersed detection histories that are poorly fit by common models at varying simulated movement levels (**A–B**, psi = 0.5, density = 0.1 individuals ha^−1^) and density levels (**C–D**, psi = 0.5, movement SD = 10 m). Rows (**A, C**) show examples of the simulated “observed” detection history for the full visit length versus the detection history expected by fit basic, Royle-Nichols (RN), and zero-inflated beta-binomial (ZIBB) occupancy models. Panels (**B, D**) show *P*-values from the goodness-of-fit test for each of 100 simulations subsampled from 2 to 20 visits per site. A low *P*-value indicates worse model fit to the observed detection history.

**FIGURE 4.**
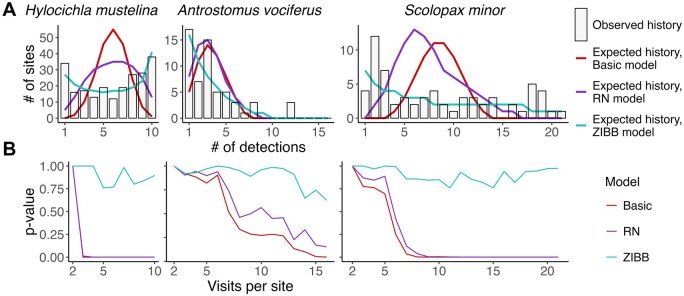
Two of three occupancy models fit field datasets poorly, but it may take many visits to identify fit issues. (**A**) Observed detection histories versus detection histories expected by fit occupancy models. (**B**) *P*-values of goodness-of-fit tests for basic, Royle-Nichols (RN), and zero-inflated beta-binomial (ZIBB) occupancy models fit to subsamples of the data from 2 to the maximum number of visits per site.

We found a signature of detection heterogeneity in 93% of the simulated full-occupancy datasets (Figure 2A; *n *= 3,000 20-visit detection histories for 160 simulated occupied sites) and in all detection histories from field data. Generally, detection heterogeneity was associated with overdispersion or “flatness” of the observed detected history compared to the expected one (Figures 3 and 4; Supplementary Material Figure 2).

Increasing sample size of occupied sites and visits per site increased the power to diagnose detection heterogeneity (Figure 2A). Among the 93% of simulated datasets with a signature of heterogeneous detection probability at the highest occupancy level, the test failed to identify heterogeneity in 35% of datasets when subset to either one or both of the lower occupancy levels (Figure 2A). In the simulated data, it took over 6 visits to identify heterogeneity in at least 50% of datasets (Figure 2A). In the field data, identifying heterogeneity took 3 visits (*H. mustelina*), 14 visits (*A. vociferus*), and 7 visits (*S. minor*) (Figure 4).

Signatures of detection heterogeneity were easiest to detect in simulations with small territory sizes (i.e., low SD of movement; Figure 2B) and low bird population density (Figure 2C). Higher perceptibility reduced the impact of territory size; rates of diagnosis of heterogeneity were nearly identical over the territory sizes we modeled when we simulated an effective detection radius (EDR) of 120 m (Supplementary Material Figure 1). Higher perceptibility was also associated with less identification of heterogeneity at higher population densities (e.g., increasing EDR to 90 m eliminated heterogeneity at a density of 2 individual ha^−1^, and increasing EDR to 120 m eliminated heterogeneity at densities of at least 1 individual ha^−1^; Supplementary Material Figure 1).

### Goodness-of-Fit Tests

#### Simulated data

We found that 2 of the 3 occupancy modeling techniques had poor fit to our simulated data (Figure 3). Basic, binomial occupancy models overwhelmingly failed goodness-of-fit tests (74% of simulations; *n *= 9,000). Royle-Nichols abundance models failed goodness-of-fit tests much less frequently than the basic occupancy model (41% of simulations), indicating that these models had better capacity to model the detection histories. In contrast, the ZIBB models never failed goodness-of-fit tests, indicating the model structure had strong capacity for modeling heterogeneous detection histories (Figure 3).

**FIGURE 5.**
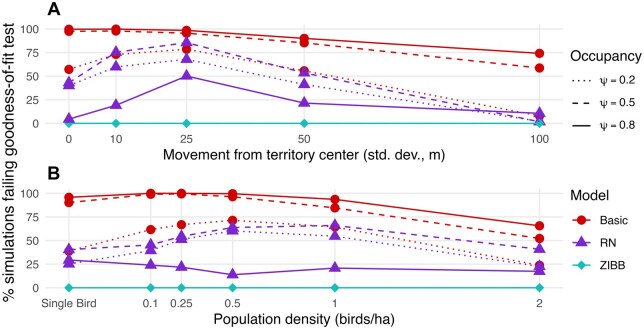
Occupancy model goodness-of-fit varies by occupancy probability and spatial arrangement of animal territories at occupied sites. Each point represents the percentage of basic, Royle-Nichols (RN), and zero-inflated beta-binomial (ZIBB) occupancy models failing the goodness-of-fit test for 100 simulated 20-visit detection histories at each value of (**A**) SD of movement away from the territory center and (**B**) density of simulated population.

Poor fit of the basic occupancy model was associated with the same conditions as detection heterogeneity (i.e., low SD of movement and low bird population density; Figures 3 and 5). In contrast, the RN model had an apparently humped relationship with movement rate and density (i.e., poor model fit was most common at low to intermediate territory sizes and most common at intermediate to high population densities; ­Figures 2 and 3).

For the basic occupancy model, the lowest occupancy probability was associated with a lower failure rate of the goodness-of-fit test. Almost all simulations with a poor fit of the basic model at lower occupancy levels also had a poor fit at higher occupancy levels. In contrast, for the RN model, the highest occupancy probability was associated with fewer failures of the goodness-of-fit test for the RN model (Figure 5; Supplementary Material Figure 3). Unlike the basic model, different simulations failed the goodness-of-fit test at different occupancy levels. For example, 60% of simulations failing the goodness-of-fit test at either of the two lower occupancy probabilities did not fail the goodness-of-fit test at the highest occupancy probability.

The power to identify poor model fit for both the basic occupancy and RN models was directly related to the number of visits per site, the impact of which varied by occupancy probability (Figure 5). For example, in simulations with an occupancy probability of 0.8, basic occupancy models failed the goodness-of-fit test after 8.8 visits on average, compared to 11.7 visits on average for simulations with an occupancy probability of 0.2.

#### Field data

The basic occupancy model failed the goodness-of-fit test for all 3 detection histories from field data, whereas the RN model failed for 2 of the 3 (*S. minor* and *H. mustelina*), and the ZIBB model never failed (Figure 4). Detection heterogeneity was diagnosed in fewer visits within the *H. mustelina* dataset (3 visits for both basic occupancy and RN models) compared to the *S. minor* dataset (7 visits under the basic model and 8 visits under the RN model) and the *A. vociferus* dataset (14 visits under the basic model).

### Occupancy Estimation Accuracy

#### Simulated data

**FIGURE 6.**
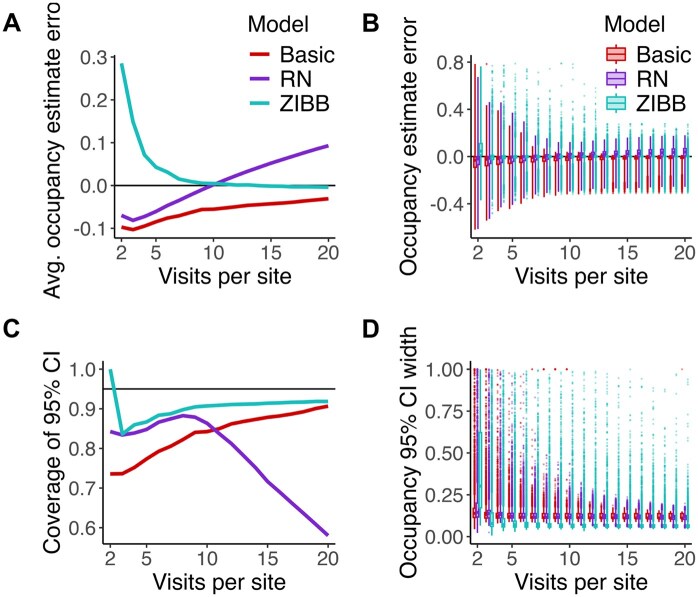
Occupancy error for basic, Royle-Nichols (RN), and zero-inflated beta-binomial (ZIBB) occupancy models fit to simulated data, calculated as the difference between model-estimated occupancy and true occupancy. (**A**) Average occupancy error for each number of visits, where the horizontal line represents zero error. (**B**) Occupancy estimate errors for each simulation. Whiskers represent the 10*IQR range, and points represent outliers beyond that range. (**C**) Coverage of the 95% CI for occupancy for each simulation, where the horizontal line represents the nominal coverage probability of 95%. (**D**) Width of the 95% CI for occupancy for each simulation.

The basic and RN models tended to produce biased estimates of occupancy (i.e., consistently over- or under-estimating occupancy), whereas the ZIBB model produced relatively unbiased estimates once fit on detection histories with enough visits per site (Figure 6). For the basic occupancy model, occupancy was most underestimated for scenarios with high true occupancy and for scenarios in which occupied sites had low densities of individual birds. For the RN model, greater overestimates were produced for scenarios in which occupied sites had higher densities of individual birds (Supplementary Material Figure 4).

The basic occupancy model consistently underestimated occupancy, but its estimates improved as the number of visits per site increased from 2 visits per site (mean error: –10% ± 34% SD) to 20 visits per site (mean error: –3% ± 9%). The RN model similarly underestimated occupancy when fit on detection histories with few visits per site (mean error, 2 visits per site: –7% ± 35%), but strongly overestimated occupancy when fit on detection histories with 10 or more visits per site (mean error, 20 visits per site: +9% ± 13%). The ZIBB model produced strong overestimates when fit on 2-visit histories (mean error: +28% ± 52%), but on average its estimates were biased 5% or less when fit on at least 5-visit histories (mean error: +4% ± 37%), achieving low bias at 20 visits (mean error: <–1% ± 12%). Excluding boundary estimates (occupancy estimate > 0.99), the ZIBB model required only 3 visits to achieve <1% bias (mean error: 1% ± 29%), whereas neither the basic nor the RN model achieved such low average bias. At worst, occupancy was underestimated by 94% and overestimated by 400%.

While the ZIBB model was least biased on average and had a smaller interquartile range of biases, the outliers in its estimates were larger than those of other models (Figure 6B). The ZIBB model was closest to achieving 95% coverage for its 95% confidence interval (Figure 6C). At low visit numbers, the ZIBB model produced larger confidence intervals for occupancy than the other models, but at higher visit numbers, its confidence intervals tended to be narrower (Figure 6D). It produced boundary estimates at a higher rate (0.6% of model fits) than the basic model (0.06%) or the RN model (0.02%), especially when fit to detection histories with fewer than 8 visits per site (Supplementary Material Figure 5).

The *P*-values of goodness-of-fit tests and detection heterogeneity tests did not meaningfully predict the magnitude of occupancy estimation error, with the notable exception of low *P*-values of the detection heterogeneity test predicting higher error in RN model fits (Supplementary Material Figure 6). As the number of visits increased, the proportion of models failing the goodness-of-fit tests increased despite increasing accuracy of occupancy estimates (Supplementary Material Figures 3 and 4).

#### Field data

**FIGURE 7.**
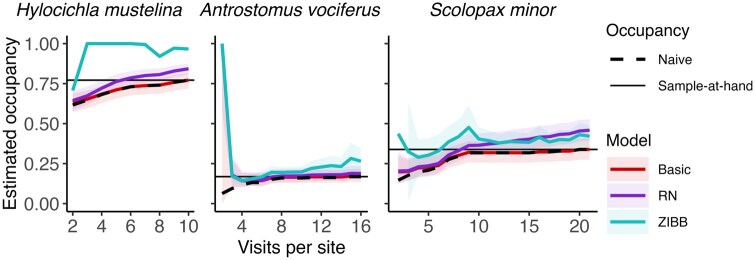
Occupancy probability (± SE) estimated by basic, Royle-Nichols (RN), and zero-inflated beta binomial (ZIBB) occupancy models on three field datasets across a range of number of visits per site. At each detection history length, graphs indicate naive occupancy, the percentage of points within a detection history that had at least one detection, and sample-at-hand occupancy, the naive occupancy for the full detection history. SEs were not calculable for some iterations of the ZIBB model.

The basic binomial occupancy model generally underestimated sample-at-hand occupancy when fit to detection histories with few visits, producing occupancy probabilities equal to the naive occupancy probability in the subsampled data (Figure 7). The RN model’s estimate was higher than the basic model’s estimate for the *H. mustelina* and *S. minor* datasets, but was nearly identical to the basic model’s estimate for the *A. vociferus* dataset. The ZIBB model’s estimates were more variable. For the *H. mustelina* dataset, the model produced boundary estimates of occupancy near 1 for most detection history lengths. For the *A. vociferus* dataset, the estimate was somewhat higher than the RN model’s estimate. For the *S. minor* dataset, the ZIBB and RN models produced similar estimates.

## DISCUSSION

Models accounting for failure to detect wildlife usually assume that the probability of detecting animals is constant across sites and visits, or is constant given site- and visit-level covariates (e.g., observer, time, vegetation, or weather). In this work, we found that processes unrelated to covariates–within-territory movement and random territory positioning–caused substantial detection heterogeneity, resulting in poor occupancy model fit and marked occupancy estimation biases for two occupancy models that assume detection probability is constant (basic occupancy model) or is constant for each individual animal at the site (Royle-Nichols model). These results suggest that detection heterogeneity may be widespread and, to a large extent, unavoidable with common sampling approaches such as point counts and passive acoustic monitoring (PAM). Furthermore, failing to diagnose and account for heterogeneity could cause incorrect ecological conclusions, such as biases in occupancy estimation at low visit numbers (e.g., average bias for basic occupancy model at two visits: 10% ± 34% SD).

While practitioners typically test for potential occupancy model misspecification, we found that tests for detection heterogeneity and poor model fit required many visits per site, had lower power at lower occupancy levels, and failed to predict occupancy estimation errors in a meaningful manner, three infrequently discussed properties of such tests (MacKenzie and Bailey 2004). Identifying detection heterogeneity required 6 visits per site on average, and identifying poor model fit required 9 visits per site, making heterogeneity and its impacts difficult to detect with the number of visits commonly included in most occupancy studies (5 or fewer per site, Goldstein et al. 2024a). Thus, practitioners working with too few visits per site may be led astray by these tests, erroneously concluding their model is well-specified. Furthermore, the *P*-values of goodness-of-fit tests did not meaningfully predict the magnitude of occupancy estimation error: occupancy estimation was most biased at low visit numbers, where tests had the least power and models were thus least likely to fail. Conversely, at high visit numbers, basic occupancy models generally failed goodness-of-fit tests despite producing negligibly biased occupancy estimates.

Detection probabilities were most likely to be heterogeneous at low density and perceptibility and, surprisingly, at smaller territory sizes or movement rates. Our study simulated a point count or autonomous recorder design in which cues produced near the survey point are more perceptible. In the extreme, when each site contains a single, stationary individual, all cues produced at a given site have a constant perceptibility. Thus, perceptibility can vary widely between sites from nearly 0 to nearly 1, with probability of detection at a given site driven primarily by the random position of the individual’s territory with respect to the survey location (Supplementary Material Figure 2). In contrast, a highly mobile individual may produce cues from a wide range of distances. While movement increases within-site variability in detection probability from visit to visit, our results suggest that movement makes the species’ detection probability more similar across sites.

Detection heterogeneity was more easily diagnosed with increasing repeat visits to each site and in higher-occupancy landscapes. Considering simulations diagnosed with heterogeneity when 80% of sites were occupied, 34% of those simulations were never diagnosed as heterogeneous when subsampled to a lower occupancy probability. Notably, less extreme heterogeneity (e.g., in scenarios with higher density or larger territory size) requires more visits to detect, but is associated with less error in occupancy estimates.

Non-constant detection typically caused modest underestimates of occupancy, but these results were model-dependent. The basic model tended to underestimate occupancy, often producing estimates near or equal to naive occupancy. As the number of visits increased, naive occupancy also increased, approaching or reaching true occupancy. Thus, basic occupancy models’ estimates improved as visit number increased, but only because naive occupancy increased–not because of improved fit or reliability (e.g., Figure 7), even if the true occupancy was much higher than the naive occupancy. Contrary to the typical expectation that detection heterogeneity results in occupancy underestimates (Royle and Nichols 2003), the RN model’s estimates began to produce strong overestimates as the number of visits increased. Similar to DiRenzo et al. (2022), the worst estimates arose from scenarios with low availability and low occupancy.

Notably, the ZIBB models produced nearly unbiased occupancy estimates when provided with at least 3 visits per site (excluding boundary estimates), and their 95% confidence intervals were better calibrated than those of the basic and RN models (Figure 6; Supplementary Material Figure 4). While the ZIBB model is capable of modeling heterogeneity, it has several potential drawbacks. First, achieving good model fit requires more visits per site, as the ZIBB model estimates three parameters, unlike the two-parameter basic occupancy and RN models. This additional flexibility comes at the cost of a larger chance of model failure, e.g., by producing more boundary occupancy estimates than the other models. Second, despite the ZIBB model having the smallest SD of error, it also produced the largest outliers (Supplementary Material Figure 4). Third, common occupancy modeling packages such as *unmarked* do not include the ZIBB model, and fitting it with covariates requires a custom implementation in a Bayesian framework such as Stan.

In light of our results, we urge caution in interpreting detection probability differences between sites as an indicator of relative site density or site usage, as some practitioners have suggested (Royle and Nichols 2003, Goldstein et al. 2024a). We found that across several levels of density and movement, the relationship between detection probability and site use or density is weak for point-count and PAM datasets, due to the strong relationship between the detectability of each individual and its position relative to the survey point. In particular, the total number of detections at each site was notably overdispersed or “flat” compared to the expectations of both typical occupancy models and, to a lesser extent, the RN model (see Methods; e.g., Figure 3, Supplementary Material Figure 2). These overdispersed detection histories suggest caution is warranted when applying and interpreting the results of approaches like the RN model, which use detection probability as a proxy for abundance.

While our analysis examines the detection homogeneity assumption, within-territory movement causing temporary emigration of an animal out of a study plot is often alternatively viewed as a violation of the closure assumption (MacKenzie et al. 2002, Chandler et al. 2011, McNeil et al. 2014, Hayes and Monfils 2015). The closure assumption states that a site’s occupancy status remains fixed between sampling occasions. Closure violations are assumed to cause inflated occupancy estimates compared to the proportion of occupied sites at any given instant (e.g., Rota et al. 2009, Otto et al. 2013; but see Kendall et al. 2013). In contrast, detection heterogeneity is often assumed to cause deflated occupancy estimates (Royle and Nichols 2003, Royle 2006). These contrasting perspectives correspond to two alternative interpretations of occupancy: “instantaneous” or “asymptotic” (Efford and Dawson 2012, Goldstein et al. 2024a). When the true occupancy state of a site is viewed as instantaneous (i.e., not varying over time), within-territory movement can cause occupancy overestimates by increasing the proportion of sites that are occupied at a given density, due to animals moving into additional study plots as time passes (Hayes and Monfils 2015). In contrast, asymptotic occupancy considers the “true” occupancy status of a site to be “the asymptote approached by cumulative observations of occupancy over time,” characterizing the occupancy state as whether or not the plot is used at all during the season (Mackenzie 2005, Efford and Dawson 2012). Our study used the asymptotic interpretation of occupancy, defining occupied sites as those where the study plot around the survey point was “used” (i.e., those at which the core of at least one individual’s territory at least partially overlapped with the circle in which the surveyor had >5% chance of detecting a single song emitted by a bird). Under this paradigm, because sites may be considered occupied even if only inhabited by distant, difficult-to-detect individuals, some level of occupancy underestimation is reasonable even with many repeat visits per site. Consistent with this definition of occupancy, we found that the basic occupancy model tended to underestimate occupancy probability.

Several assumptions in our simulations may limit the generality of our results. First, we decoupled the occupancy and density processes to investigate separately their influence on the presence, diagnosis, and model-fitting impacts of detection heterogeneity. In each simulation, we selected a proportion of sites to simulate as occupied, then simulated each occupied landscape with the same density of individual birds. We ensured that each occupied site at least partially overlapped the core territory of at least one individual bird. Thus, the simulated density only impacted detection probability, not occupancy probability. The basic occupancy model similarly assumes that occupancy and detection are separate processes, allowing for any combination of occupancy probability and detection probability. In contrast, the RN model assumes both occupancy and detection are impacted by density or abundance. The RN model uses a Poisson distribution to account for the differences in detection probability arising due to abundance differences between sites. Simultaneously, the Poisson distribution models the probability that a site is unoccupied as the probability of drawing an abundance of zero from the distribution. Thus, the RN model is potentially less flexible for fitting the arbitrary percentage of unoccupied sites we simulated.

Second, while we sought to select a range of realistic parameters for animal behavior, availability for detection, perceptibility, occupancy, and density (Figure 3), we did not test the entire range of plausible parameters. Our results suggest that detection heterogeneity will be less prevalent as density, territory size, and perceptibility increase above the maximum values we simulated (2 individuals ha^−1^, 12.6-ha core territory size, 120-m EDR). We did not vary other parameters that impact availability (e.g., vocalization rate), but factors that increase availability should decrease detection heterogeneity (DiRenzo et al. 2022). We selected parameters based on estimates for point counts by Edwards et al. (2023) for North American forest songbirds, but parameters vary widely between species and differ between point count and PAM datasets (Knight et al. 2025). However, the point count simulator *bSims* created detection histories comparable to the many-visit histories generated through PAM data analyzed with machine learning classifiers and reviewed for accuracy. A fruitful area of future research would be to assess the extent to which fixed-radius point counts (Ralph et al. 1995) or amplitude-based radius truncation for PAM (Lebeuf-Taylor et al. 2025) could reduce heterogeneity.

Third, we did not investigate variation in occupancy and detectability caused by covariates, nor did we include covariates in our models. While we intentionally simulated data without detection or occupancy covariate effects, our field PAM data likely contained unmodeled effects. In particular, our *H. mustelina* dataset originates from R. P. Lyon et al. (personal communication), who modeled it using the ZIBB model due to detection heterogeneity under the basic occupancy model, but also found evidence for additional detection covariates. Similarly, our *S. minor* dataset had not been analyzed previously, but sky condition, temperature, and wind all impact the detectability of singing *S. minor* (Blankenship 1957, McNeil et al. 2023). Generally, we intend our field datasets to illustrate how heterogeneity may be severe when unmodeled, and how PAM can provide the many-visit detection histories required to identify heterogeneity and model it successfully. However, in practice, visits should be removed from PAM datasets when they do not meet standard survey protocols due to factors such as wind, precipitation, temperature, or nonindependence of closely spaced visits (Ralph et al. 1995, U.S. Fish and Wildlife Service 2024). Furthermore, even after removing inappropriate survey days and accounting for covariates, it would be challenging to attribute additional heterogeneity to within-territory movement over other sources, e.g., closure violations or individual differences. A fruitful area for future research would be understanding the relative impact of these sources of detection heterogeneity, possibly with the use of telemetry (e.g., Valente et al. 2024) or acoustic localization arrays for individual behavior monitoring (Rhinehart et al. 2020).

The increased temporal coverage of PAM may enable better modeling not only of detection heterogeneity, but also of animal behaviors that violate the assumptions of occupancy and related models. For example, closure may be violated due to distribution dynamics (e.g., site abandonment, territory shifts, birth or death; Valente et al. 2017). Such distribution dynamics may become more prevalent when using longer sampling seasons, such as point-count surveys separated by multiple weeks or PAM deployed for an entire breeding season. To estimate distribution dynamics, many authors advocate estimating detection probability by resurveying a site multiple times in rapid succession, then estimating occupancy changes using surveys separated by longer periods of time (a “robust design,” Pollock 1982, Rota et al. 2009, Kendall et al. 2013). However, rapidly repeated surveys are more likely to be autocorrelated, producing, for example, a series of correlated nondetections due to temporary emigration, or a series of correlated detections due to a singing bout. Correlated nondetections may be incorrectly interpreted as site extinction or abandonment (Valente et al. 2017), potentially leading to inaccurate conclusions about dispersal or habitat quality (e.g., Betts et al. 2008). Similarly, correlated detections may also cause detection probability to be overestimated, resulting in underestimation of occupancy (Goldstein et al. 2024b). Passive acoustic monitoring can improve the ability to select independent samples without compromising the number of sites surveyed, as it can generate several hours of monitoring per day over multiple weeks, which can then be subsampled (Fiss et al. 2025).

### Conclusion

We found that random spatial processes, like random placement of territories and within-territory movement, cause significant differences in animals’ detection probabilities among sites. Our results suggest that, in point count and passive acoustic monitoring datasets, detection heterogeneity is likely widespread across bird populations with small territories or display sites (e.g., <3 ha) or low densities (e.g., <1 individual ha^−1^). Detection heterogeneity may also arise in any sampling approach where animals move out of fixed sampling areas, such as amphibian/reptile cover boards or game camera monitoring.

Furthermore, the number of visits strongly impacts occupancy estimation accuracy, including whether occupancy is underestimated or overestimated, potentially undermining the comparability of monitoring programs conducted with different numbers of visits. Such differences could undermine the purpose of occupancy surveys, including estimating local population size, understanding how environmental covariates impact density, and identifying the relative suitability of sites studied in different surveys.

Our results have practical implications for both avian point count monitoring and PAM surveys. We suggest practitioners use caution in interpreting results of detection heterogeneity or goodness-of-fit tests, as their power to identify poor model fit is directly related to the number of visits per site. When models were fit to detection histories with more visits per site, they tended to fail goodness-of-fit tests more frequently, despite estimating occupancy better. For systems such as the forest songbird species we modeled, the number of site visits required to diagnose heterogeneity, identify goodness-of-fit issues, or accurately model occupancy is challenging to predict. For the best chance of addressing detection heterogeneity, practitioners should conduct as many surveys as is feasible. Performing enough in-person point counts to have a reasonable expectation of diagnosing and accounting for detection heterogeneity may require prohibitive amounts of effort. In contrast, PAM can enable conducting more surveys per site with less additional field effort, facilitating the diagnosis of detection heterogeneity, improving occupancy estimation, and enabling the use of more flexible occupancy models such as the ZIBB model.

## Supplementary Material

duag006_Supplementary_Data

## Data Availability

Analyses reported in this article can be reproduced using the data and code provided by Rhinehart et al. (2026).
