# Supplementary material for: Addressing widespread detection heterogeneity in avian occupancy modeling using passive acoustic surveys
Source: Condor. 2026 Jan 22;128(2):1–15. doi: 10.1093/ornithapp/duag006 (PMC13168550; doi:10.1093/ornithapp/duag006)
Supplement: duag006_Supplementary_Data [file duag006_supplementary_data.pdf]

Supplementary Material

Supplementary Methods - Passive acoustic monitoring data collection and analysis

American Woodcock (Scolopax minor)

Fieldwork was conducted across Pennsylvania, U.S., within the Appalachian Mountains bird conservation region (Kelley et al 2008). Survey locations were chosen within regenerating secondary forest following management. Elevation ranged from 189 to 879 meters above sea level across sites. We expected to find *S. minor* in these areas as they use young forests with dense understory for foraging and nesting habitat and utilize nearby open or shrubby habitat for singing grounds where males give audible peent calls and perform flight displays (Macaulay et al 2020). The peent call is diagnostic and is used to confirm *S. minor* presence on breeding grounds following the USFS American Woodcock Singing Ground Survey (SGS) protocol (USFWS).

We used Audiomoth 1.1.0 (Open Acoustic Devices) units running firmware 1.5.0 to record soundscapes. Units were programmed to record daily from 2030 to 2200 Eastern Daylight Time using a sample rate of 32kHz and medium gain. Recorders were deployed roughly 1.5m above the ground in anti-static ziplock bags along with a desiccant pack within a camouflaging brown mesh bag. Recorders were deployed in early April and retrieved after they had run out of battery in late June.

We restricted our analyses to recordings occurring within the SGS period, a 38-minute window starting 22 minutes after sunset on evenings with <75% cloud cover or starting 15 minutes after sunset when cloud cover is greater. To account for the lack of ability to determine cloud cover from our acoustic data, we used the later start time and earlier end time, resulting in a 31-minute daily survey window.

As recorders were all programmed with an identical recording schedule that did not vary with sunset, the number of minutes from each recorder that overlapped with our survey window varied by date and location. Earlier recording dates only captured the end of the survey window, while later dates captured most or all of it. We removed recorders without overlap with the SGS survey window, leaving a total of 135 unique survey locations. Overall, 67,237 minutes were analyzed, averaging 23.7 minutes per location per day.

The *S. minor* peent call (right) consists of a short, buzzy phrase with fast amplitude modulation somewhat similar to a frog call. Its consistent pulse rate, duration, and frequency range allowed us to create an automated detector using the RIBBIT method

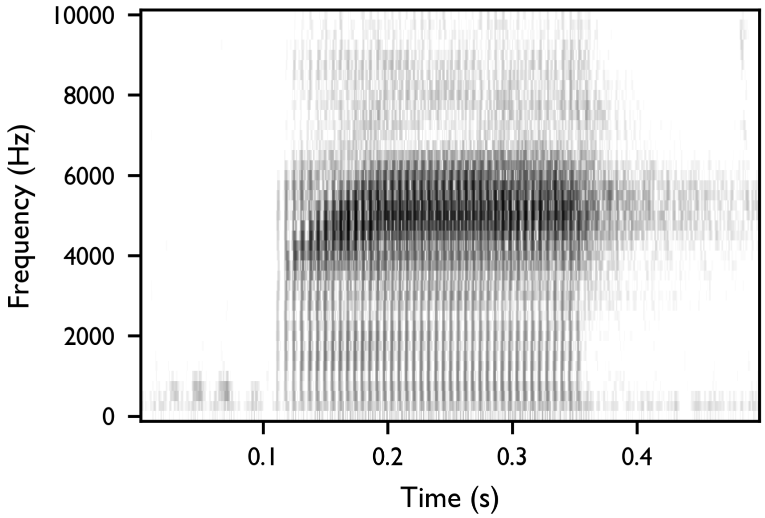

in which these audio parameters are used to score audio clips from field recordings for the likelihood of containing a target sound (Lapp et al 2021). The parameters used for RIBBIT were a pulse rate of 140,200, a signal frequency range of 4000-6000 Hz, and a clip duration of 0.5 seconds with 0.25 seconds of overlap to account for the possibility of a peent call occurring directly on a clip border. We included a noise band from 0-1000 Hz to help filter out environmental sounds such as wind and rain that might result in high-scoring clips containing no target vocalizations.

After running our detector on all field recordings occurring within the survey window, we extracted the top 5 highest-scoring 0.5-second clips for each day from April 15 to May 5 from each recorder. These clips were manually reviewed by an expert to confirm potential peent call detections. If at least one peent call was detected across these five clips, that particular site/day combination was marked with a 1 for a detection; otherwise, it was marked as a 0 if no peent calls were detected. This produced a confirmed 21-day detection history for each site.

Eastern Whip-poor-will (Antrostomus vociferus)

AudioMoth (v1.0/1.1) autonomous recording units (Hill et al. 2018) were deployed at 350 locations across south-central, southwestern, and northeastern Pennsylvania on public forested lands in 2022. All ARUs were at least 250 m apart and strapped to <10 cm DBH trees approximately 1 m from the ground in

plastic ziploc bags with a small desiccant packet. These ARUs recorded at a sample rate of 32 kHz from 9:30-11:00 PM EDT between June 7 and Jun 22, encompassing part of the window of time in which *A. vociferus* surveys could be conducted in this year. The potential survey window extends beyond the end date but batteries were exhausted after June 22. For more information on appropriate acoustic-based surveys for this species, see the Nightjar Survey Network (nightjars.org).

The classifier used to process acoustic data was created in Python using the package OpenSoundscape (v0.6.1; Lapp et al. 2023) with a Resnet18 architecture and pre-trained weights from ImageNET (Deng et al. 2009). We chose to structure classes as binary and single-target (one class for *A. vociferus* song and one class for other sounds). The classifier was trained on five-second samples of the species' songs obtained from Xeno-canto in 2020. To test the trained model's performance, we used a set of 3000 clips obtained from AudioMoth recorders collected across temperate forests spanning from North Carolina to Maine and annotated for the presence of *A. vociferus* songs. We then evaluated precision and recall across score thresholds on these data. Remarkably, the model reached a precision of 100% (no false-positives) at relatively high values of recall (percentage of all clips containing *A. vociferus* song considered to be true positives at this threshold) using scores from the *A. vociferus* song class. Ultimately, we chose a threshold of 4.3 where all clips scored at least as high as 4.3 were considered true positives. At this threshold, precision was 100% and recall 35% (meaning all clips above the threshold should contain *A. vociferus*, but the protocol misses about 65% of all clips containing *A. vociferus* songs). For further information on classifier training and the context surrounding the creation of this classifier, see Larkin et al. 2024.

The classifier was applied to ARU data split into five-second clips to produce scores. Detection histories were assembled from scores associated with data from survey location-date combinations. When any five-second clip from one survey location-date combination received a score of at least 4.3, that survey location-date combination received a "1," indicating that *A. vociferus* was present. All other survey location-date combinations received a "0," indicating that *A. vociferus* was not detected. This produced 16-day detection histories for each recording location.

Wood Thrush (Hylocichla mustelina)

AudioMoth (v1.0/1.1) autonomous recording units (Hill et al. 2018) were deployed at 668 sampling points within six study areas across southwestern and central Pennsylvania, USA, in 2020 and 2021. These units recorded the avian community from May 15th through June 30th. For the purposes of this study, we used data recorded from two areas, specifically Laurel Ridge and Ohiopyle State Parks. Ohiopyle and Laurel Ridge State Parks consisted of 153 and 139 sampling points, respectively. Each unit recorded for two hours from 6 am to 8 am EDT throughout the entire deployment period. AudioMoth recorders were programmed to record each day using a 32 kHz sampling rate and on the medium gain setting. AudioMoths were deployed on tree trunks <26 cm diameter at breast height and approximately 1.5-2.0 meters off of the ground to avoid issues with acoustic masking. Each AudioMoth was deployed in an anti-static waterproof bag with a desiccant pack placed behind the device.

We developed a convolutional neural network (CNN) multi-species automated birdsong classifier using OpenSoundscape (Lapp et al. 2023) to detect birdsong within recordings for 3 eastern forest songbirds, including *H. mustelina*. We trained this CNN classifier using publicly available song recordings from Xeno-Canto (https://xeno-canto.org). We used Raven Software (Raven Pro 1.6.4, Bioacoustics Research Program 2022) to generate time and frequency boundaries with annotations for songs from Xeno-Canto recordings for each species to generate training clips. To prepare training clips for the classifier, we followed protocols from Chronister et al. (2025).

After running our CNN classifier on all field recordings occurring within the survey window, we extracted the single highest-scoring 4-second clip for each day from May 22nd - 31st 2020 and 2021 from each recorder. In short, these clips were manually reviewed by expert listeners to confirm detection of *H. mustelina* songs. Site/day combinations were marked with a 1 for a confirmed detection, or a 0 for a nondetection. This produced a confirmed 10-day detection history for each site.

Supplementary figures

Larger versions of all figures can accessed on Dryad at doi.org/10.5061/dryad.djh9w0wdr

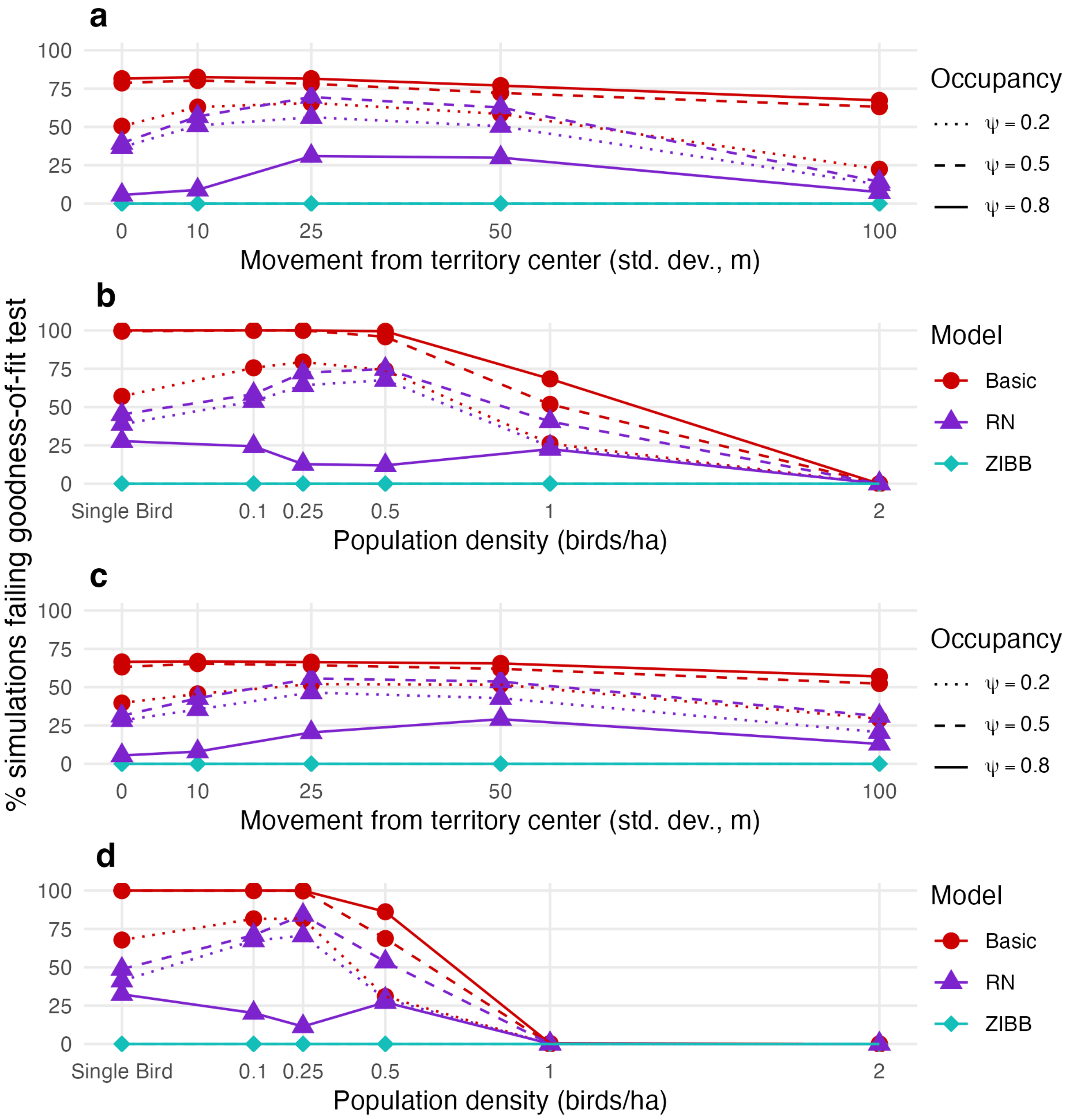

Figure S1. Percentage of simulation scenarios assessed to have unmodeled detection heterogeneity under each occupancy model type and percentage of occupied sites for a simulated effective detection radius of 90 m (a-b) and 120 m (c-d). Results subset by standard deviation of movement away from territory center (a, c) and simulated population density (b, d).

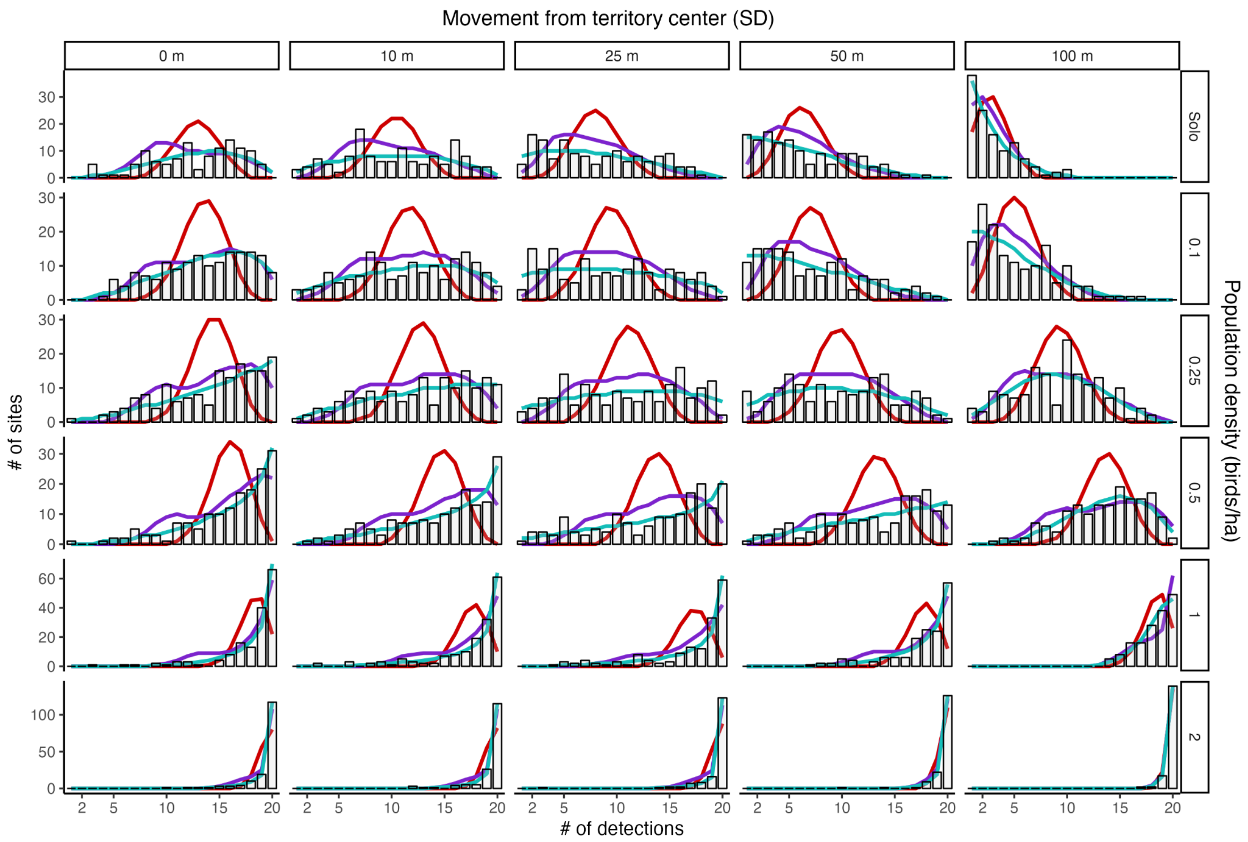

Figure S2. Observed distribution of detections per site versus distributions expected by the basic occupancy model, Royle-Nichols (RN) model, and zero-inflated beta-binomial (ZIBB) model. One out of the 100 simulations is shown for each of the 30 scenarios simulated at occupancy = 0.8.

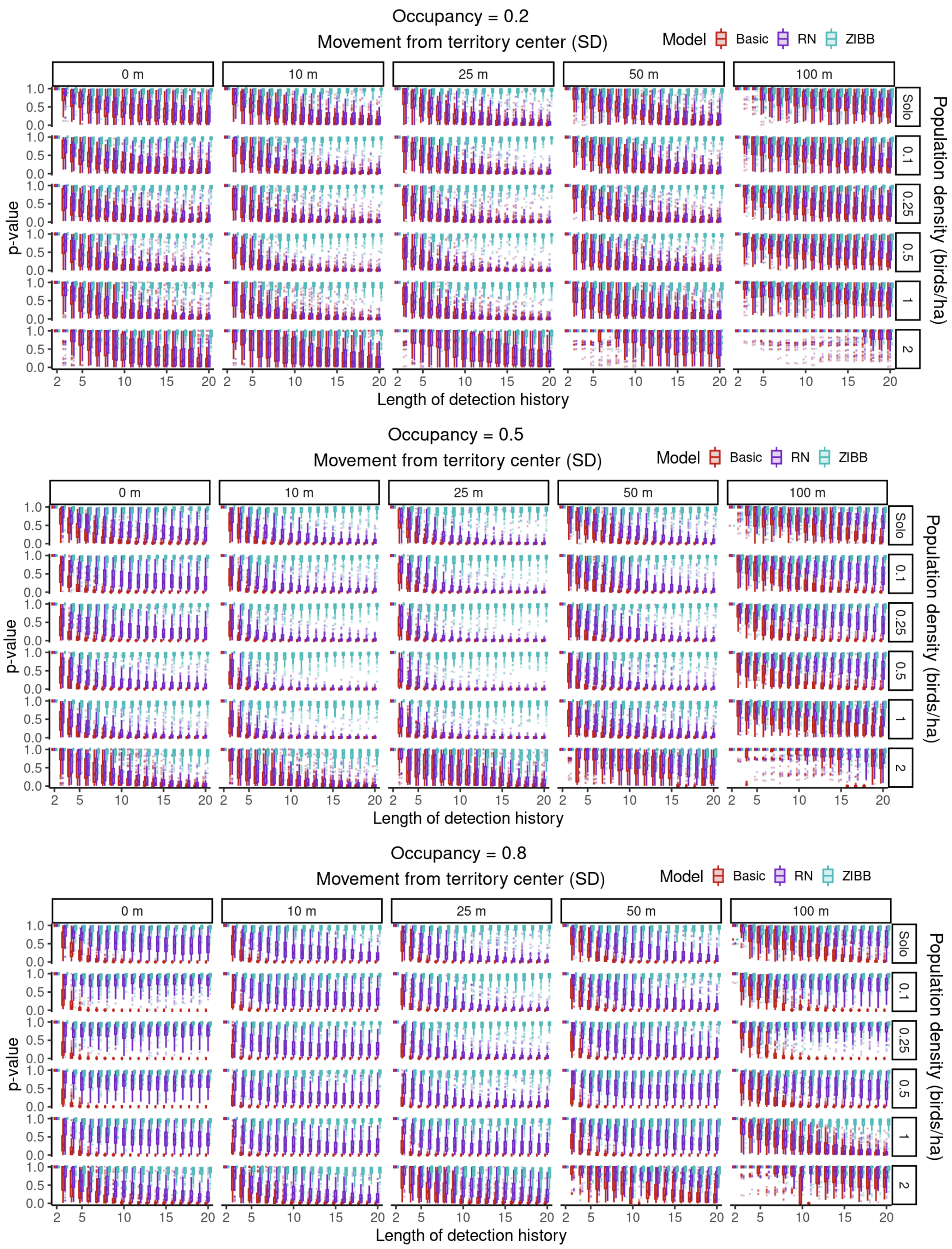

Figure S3. Results of goodness-of-fit tests to diagnose model misspecification for 30 scenarios simulated at 20%, 50%, and 80% occupancy. Lower *p* values imply greater discrepancies between the observed detection history and the detection history expected under a given model (basic; Royle-Nichols, RN; or zero-inflated beta binomial, ZIBB).

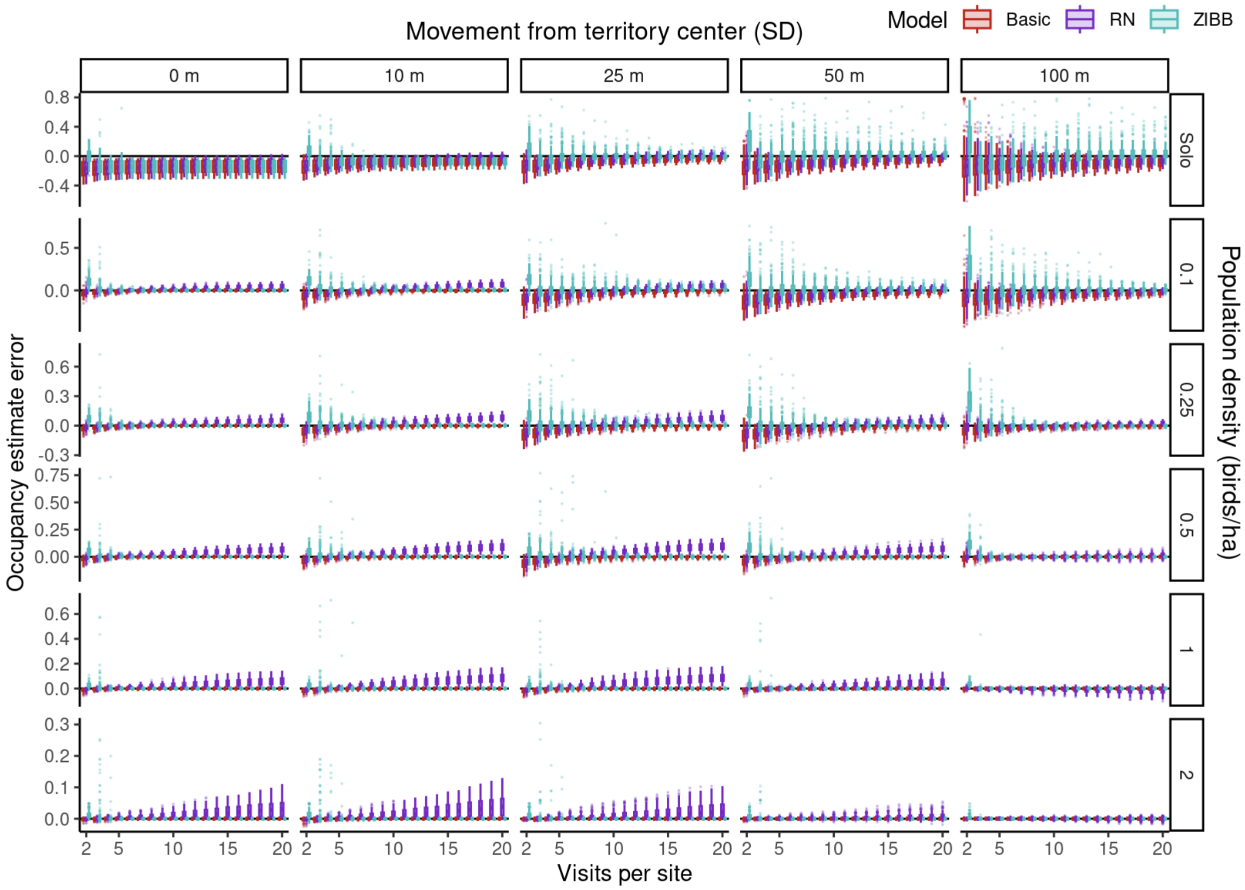

Figure S4. Biases in occupancy across 300 simulations for each of 30 simulated scenarios. Each panel shows the differences between estimated and true occupancy for one of three occupancy models, a basic occupancy model, the zero-inflated beta-binomial (ZIBB) model, or the Royle-Nichols (RN) model).

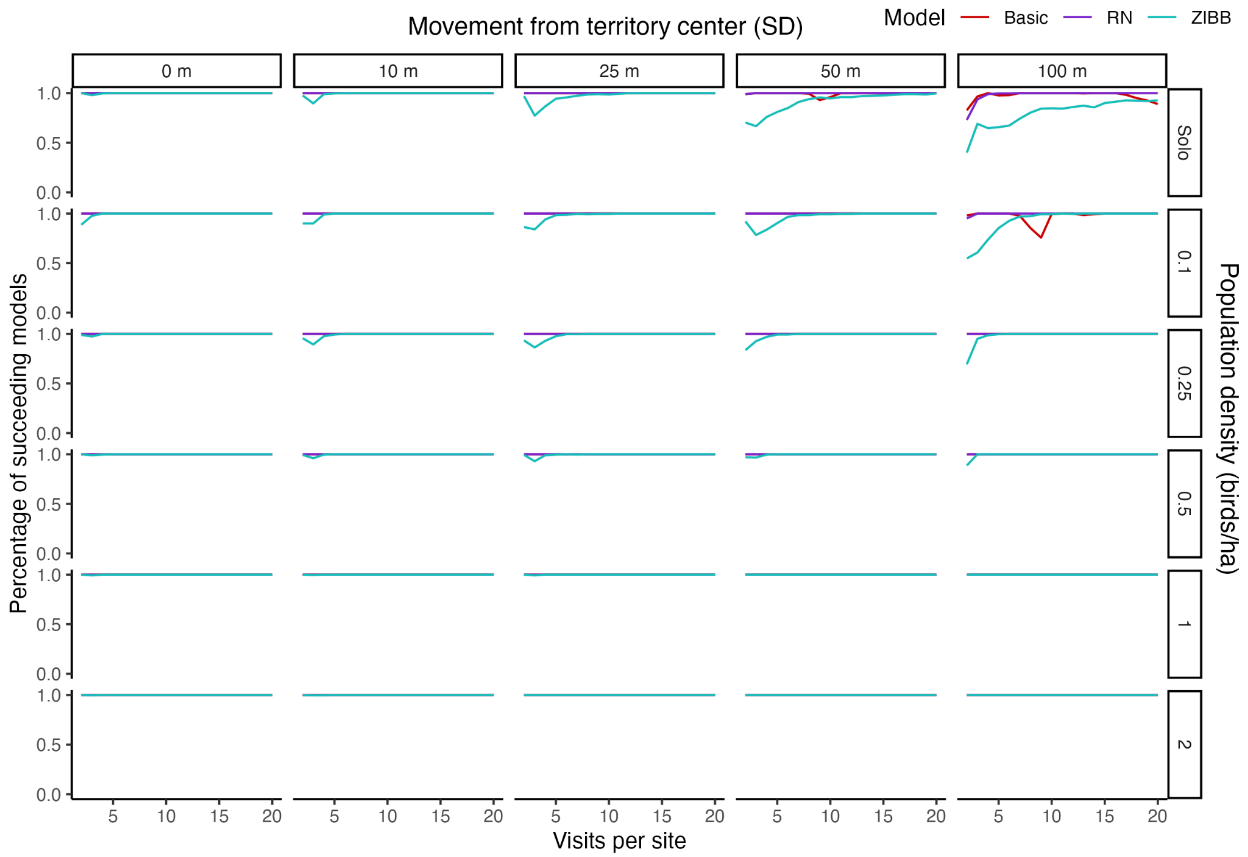

Figure S5. Percentage of simulations that succeeded (i.e., were not removed due to boundary estimates of occupancy >= 0.99) for 300 simulations of each of 30 scenarios for basic, Royle-Nichols (RN), and zero-inflated beta-binomial (ZIBB) occupancy models

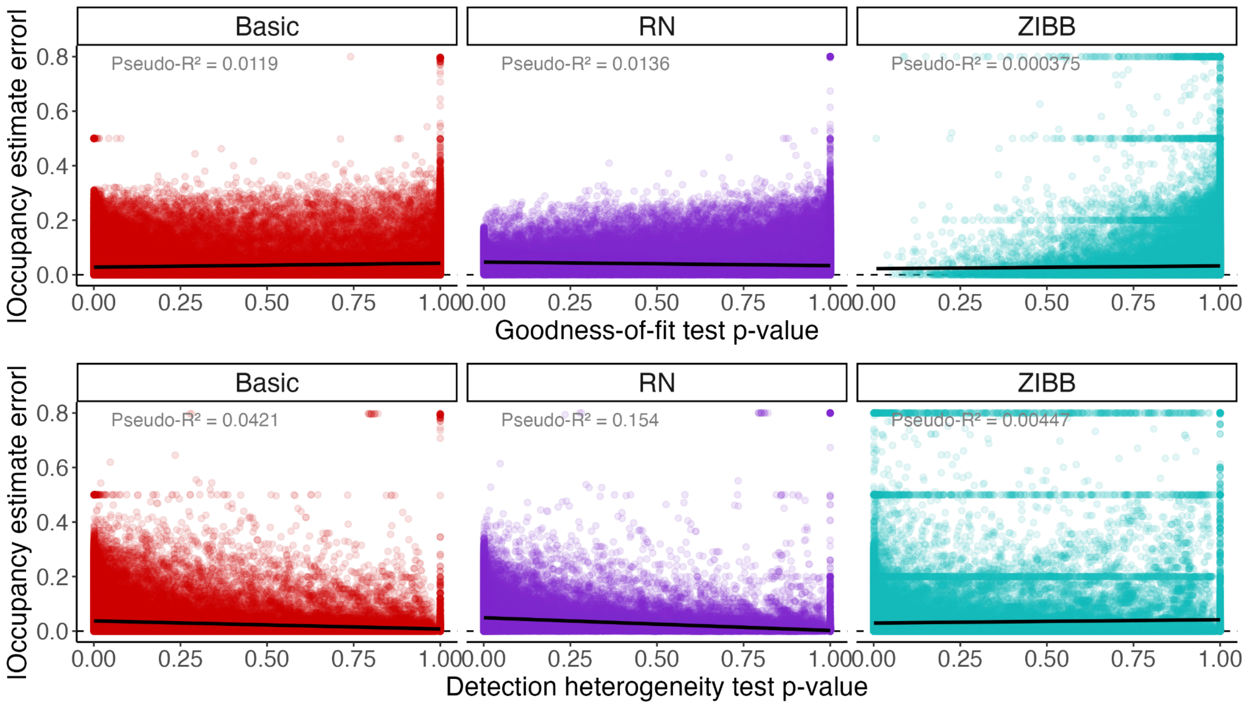

Figure S6. Goodness-of-fit test and detection heterogeneity test *p* values are not strongly related to the magnitude of occupancy estimation errors for basic, Royle-Nichols (RN), and zero-inflated beta-binomial (ZIBB) occupancy models. McFadden's pseudo-R^2 calculated from a quasi-binomial GLM is shown.
